# Preimplantation genetic diagnosis associated to Duchenne muscular dystrophy

**DOI:** 10.1590/S1679-45082017RC3994

**Published:** 2017

**Authors:** Bianca Bianco, Denise Maria Christofolini, Gabriel Seixas Conceição, Caio Parente Barbosa

**Affiliations:** 1 Faculdade de Medicina do ABC, Santo André, SP, Brazil.

**Keywords:** Muscular dystrophy, Duchenne, Polymerase chain reaction, Comparative genomic hybridization, Prenatal care, Case reports, Distrofia muscular de Duchenne, Reação em cadeia da polimerase, Hibridização genômica comparativa, Cuidado pré-natal, Relatos de casos

## Abstract

Duchenne muscular dystrophy is the most common muscle disease found in male children. Currently, there is no effective therapy available for Duchenne muscular dystrophy patients. Therefore, it is essential to make a prenatal diagnosis and provide genetic counseling to reduce the birth of such boys. We report a case of preimplantation genetic diagnosis associated with Duchenne muscular dystrophy. The couple E.P.R., 38-year-old, symptomatic patient heterozygous for a 2 to 47 exon deletion mutation in *DMD* gene and G.T.S., 39-year-old, sought genetic counseling about preimplantation genetic diagnosis process. They have had a 6-year-old son who died due to Duchenne muscular dystrophy complications. The couple underwent four cycles of intracytoplasmic sperm injection (ICSI) and eight embryos biopsies were analyzed by polymerase chain reaction (PCR) for specific mutation analysis, followed by microarray-based comparative genomic hybridisation (array CGH) for aneuploidy analysis. Preimplantation genetic diagnosis revealed that two embryos had inherited the maternal *DMD* gene mutation, one embryo had a chromosomal alteration and five embryos were normal. One blastocyst was transferred and resulted in successful pregnancy. The other embryos remain vitrified. We concluded that embryo analysis using associated techniques of PCR and array CGH seems to be safe for embryo selection in cases of X-linked disorders, such as Duchenne muscular dystrophy.

## RESUMO

A distrofia muscular de Duchenne é a doença muscular mais comum observadas em crianças do sexo masculino. Atualmente, não há terapia eficaz disponível para distrofia muscular de Duchenne, portanto, é essencial o diagnóstico pré-natal e o aconselhamento genético para reduzir o nascimento desses meninos. Relatamos um caso de diagnóstico genético pré-implantação associado à distrofia muscular de Duchenne. O casal E.P.R., 38 anos, heterozigota, sintomática para uma mutação de deleção dos éxons 2 a 47 no gene *DMD* e G.T.S., 39 anos, buscaram aconselhamento genético sobre o processo de diagnóstico genético pré-implantação. O casal relatou que tiveram um filho de 6 anos que morreu devido a complicações da distrofia muscular de Duchenne. Os pacientes realizaram quatro ciclos de injeção intracitoplásmica de espermatozoides (ICSI) e oito biópsias de embriões foram analisadas por reação em cadeia da polimerase (PCR) para análise de mutação específica, seguida hibridação genômica comparativa baseada em microarranjos ( *array* CGH) para a pesquisa de aneuploidias. O diagnóstico genético pré-implantação revelou que dois embriões haviam herdado a mutação materna no gene *DMD* , um embrião tinha uma alteração cromossômica e cinco embriões eram normais. Um blastocisto foi transferido e resultou em gravidez bem sucedida. Os outros embriões permanecem vitrificados. Concluímos que a análise de embriões utilizando técnicas associadas de PCR e CGH *array* mostrou-se segura para a seleção de embriões em casos de doenças ligadas ao X, como a distrofia muscular de Duchenne.

## INTRODUCTION

Duchenne muscular dystrophy (DMD; MIM 310200) is an X-linked recessive disorder that is caused by mutation of the *DMD* gene (gene ID: 1756) located at Xp21.^( 1 )^ Duchenne muscular dystrophy is the most common muscle disease in male children, with an incidence of 1:3,500 live born males.^( 1 )^ The dystrophin gene is the largest identified human genes. It contains 79 exons, at least seven different tissue-specific promoters and two polyadenylation sites. However, dystrophin RNA is differentially processed producing a variety of transcripts encoding a set of protein isoforms.^( 1 - 3 )^ The protein translated from the larger transcript is an important cytoskeletal protein, which helps the cytoskeleton of each muscle fiber to connect to the underlying basal lamina. Alteration or loss of dystrophin forces excessive amounts of calcium into the cell membrane, resulting in excess water formation in the mitochondria; thus, the affected skeletal muscle will result in dystrophy, mitochondrial dysfunction, and necrosis.^( 4 )^

The majority of identified mutations are deletions, accounting for approximately 60-65% of DMD. Duplications have been observed in 5-15% and the remaining cases and they might be caused by small mutations such as microdeletions, microinsertions, point mutations, or splicing mutations.^( 1 , 2 , 5 )^ Approximately one-third of the DMD patients originate through new mutations, while the rest are inherited through carrier mothers or arise from germ line mosaicism.^( 6 , 7 )^

Pathologically, DMD is characterized by rapidly progressive degeneration and necrosis of the proximal muscles and calf pseudo-hypertrophy. Most DMD patients show muscle weakness in early childhood, become wheelchair-dependent by around 12 years old, and die for respiratory or cardiac failure in the late adolescence or early 20s. Currently, there is no effective therapy available for DMD patients. Therefore, it is essential to make a prenatal diagnosis and provide genetic counseling to reduce birth of such boys.^( 4 )^

We report a case of preimplantation genetic diagnosis associated with Duchenne muscular dystrophy.

## CASE REPORT

E.P.R., 38yo, symptomatic patient heterozygous for a 2 to 47 exon deletion mutation in *DMD* gene and G.T.S., 39yo, sought genetic counseling service at the *Instituto* *Ideia Fertil* , *Centro de Reprodução Humana* *e Genética* *of Faculdade de Medicina do ABC* , Santo André, São Paulo, Brazil in February 2014 to know about the preimplantation genetic diagnosis (PGD) process. They have had a 6-year-old son who died in 2012 due to DMD complications. The couple underwent four cycles of assisted human reproduction treatment with intracytoplasmic sperm injection (ICSI). Ovarian stimulation was performed by using 200UI of exogenous recombinant follicle stimulating hormone from the second day of the menstrual cycle. When the largest follicle reached 14mm, the antagonist of gonadotropin-releasing hormone (GnRH) was also administrated. After, when the largest follicles reached 17mm, human chorionic gonadotropin (hCG) a dose of 5000IU was provided and 36h later the oocyte retrieval was performed. Eight D5/D6 embryos biopsies were analyzed by PCR for specific mutation analysis, followed by array CGH for aneuploidy analysis (screening of 24 chromosomes) in a specialized private laboratory.

The genetic analysis disclosed that two embryos had inherited the maternal *DMD* mutation, one embryo had a chromosomal alteration [47,XY,del(8)(q24.11-qter),+18] and five embryos (three males and two females) were normal. One blastocyst was transferred and resulted in successful pregnancy. The child was a girl, born after 38 gestational weeks by cesarean section, weighting 2.970kg and measuring 43cm. The other embryos remained vitrified.

## DISCUSSION

The majority (>90%) of women with mutations in the *DMD* gene is asymptomatic. Although, the mutation carrier woman may be affected by DMD as a result of changes in the pattern of X chromosome inactivation, Turner syndrome (45,X) or translocation of X chromosome to an autosome. Women with muscular dystrophy symptoms are often considered as limb-girdle muscular dystrophy patients.^( 6 )^ So far, there are few population data on the prevalence of carriers, especially on symptomatic carriers. The knowledge about the condition of carrier pathogenic mutation is important to the genetic counseling, mainly because half of the children of carriers should be affected by this disease and half of the daughters will be the bearer of the pathogenic mutation.

The DMD prevention importance has been widely emphasized, once there is no curative therapy available. For those women at risk, genetic counseling can be offered and also alternatives to prevent transmission of the mutated allele, such as carrier screening tests, human assisted reproduction with PGD or the use of donor eggs and prenatal diagnosis.^( 8 )^ The PGD to determine just sex is not enough, especially in our case because the girl could be a carrier of the mutated allele and develop symptoms of DMD, as well as her mother. A male embryo is tested by direct analysis of familial mutation to determine if he is affected. The birth of affected boys can be prevented by terminating the pregnancy in countries where abortion is allowed. In these cases, parents need to face the emotional burden for the possibility to abort a healthy boy, a fact that is the major drawback of prenatal diagnosis of X-linked disorders by sex determination only. By using the PGD, either unaffected female or male embryos can be selected and transferred to the uterus.^( 8 )^ It is important to emphasize that perform PGD in cases of monogenic diseases, it is essential to know the mutation, since the genetic analysis is performed on a small number of cells and the PCR is specific for the mutation of the family being studied. Therefore, if the family does not have the molecular diagnosis, it is necessary to study the familial mutation beforehand by ligation analysis. Furthermore, with the knowledge of the specific familial mutation and options to prevent the birth of affected boys, offer a couple the chance to plan their reproductive future.

In conclusion, the embryo analysis using associated techniques of PCR and array CGH showed to be safe for embryo selection in cases of X-linked disorders, such as Duchenne muscular dystrophy.
